# Inducible Clindamycin Resistance and Biofilm Production among Staphylococci Isolated from Tertiary Care Hospitals in Nepal

**DOI:** 10.3390/idr13040095

**Published:** 2021-12-07

**Authors:** Sarita Manandhar, Raju Shrestha, Ratna Shova Tuladhar, Sunil Lekhak

**Affiliations:** 1 Department of Microbiology, TriChandra Multiple College, Tribhuvan University, Kathmandu 44600, Nepal; ratna.tuladhar007@gmail.com; 2 Department of Microbiology, National College, Khushibun, Kathmandu 44611, Nepal; shrestharaj10@yahoo.com; 3 Decode Genomics and Research Center, Sinamangal, Kathmandu 310327, Nepal; Lekahk.s@hotmail.com

**Keywords:** staphylococci, MLS_B_, *ica* genes, biofilm

## Abstract

Resistance to antibiotics, biofilm formation and the presence of virulence factors play important roles in increased mortality associated with infection by staphylococci. The macrolide lincosamide streptogramin B (MLS_B_) family of antibiotics is commonly used to treat infections by methicillin-resistant isolates. Clinical failure of clindamycin therapy has been reported due to multiple mechanisms that confer resistance to MLS_B_. This study aims to find the incidence of different phenotypes of MLS_B_ resistance and biofilm production among staphylococci. A total of 375 staphylococci were isolated from different clinical samples, received from two tertiary care hospitals in Nepal. Methicillin resistance was detected by cefoxitin disc diffusion method and inducible clindamycin resistance by D test, according to CLSI guidelines. Biofilm formation was detected by the tissue culture plate method and PCR was used to detect *ica* genes. Of the total staphylococci isolates, 161 (42.9%) were *Staphylococcus aureus,* with 131 (81.4%) methicillin-resistant strains, and 214 (57.1%) isolates were coagulase-negative staphylococci, with 143 (66.8%) methicillin-resistant strains. The overall prevalence of constitutive MLS_B_ (cMLS_B_) and inducible MLS_B_ (iMLS_B_) phenotypes was 77 (20.5%) and 87 (23.2%), respectively. Both iMLS_B_ and cMLS_B_ phenotypes predominated in methicillin-resistant isolates. The tissue culture plate method detected biofilm formation in 174 (46.4%) isolates and *ica* genes in 86 (22.9%) isolates. Among biofilm producing isolates, cMLS_B_ and iMLS_B_ phenotypes were 35 (20.1%) and 27 (15.5%), respectively. The cMLS_B_ and iMLS_B_ were 11 (12.8%) and 19 (22.1%), respectively, in isolates possessing *ica* genes. Clindamycin resistance in the form of cMLS_B_ and iMLS_B_, especially among MRSA, emphasizes the need for routine D tests to be performed in the lab.

## 1. Introduction

*Staphylococcus aureus* (*S. aureus*) and coagulase-negative staphylococci (CNS) are recognized as common causes of nosocomial and community-acquired infections worldwide. They cause a broad spectrum of diseases from mild skin and soft tissue infections to life-threatening sepsis, pneumonia, endocarditis and deep-seated abscesses [1,2]. Resistance to multiple antibiotics and the presence of virulence factors play important roles in increased mortality associated with staphylococci infection. In addition to extracellular toxins and surface structures that are effective in the induction and continuance of infection in the host, the ability to form biofilm is an important complicating factor associated with many chronic infections [3]. Biofilms are communities of bacteria, immobilized by an extracellular polymeric matrix, which can bind to biotic and abiotic surfaces. Several genes are involved in the formation of biofilm by staphylococci. The intercellular adhesion operon (*ica*) comprising *icaADBC* genes encodes polysaccharide intercellular adhesion (PIA), the main component of exopolysaccharide matrix surrounding the bacterial cells within the biofilm. Bacteria, protected by biofilm, are resistant not only to host defense mechanisms but also to standard antibiotic therapy [4,5].

The increasing rate of drug resistance and emergence of methicillin-resistant strains, for which limited treatment options exist, have created a great problem in the management of infections caused by staphylococci. This has impelled the usage of macrolide–lincosamide–streptogramin B (MLS_B_) antibiotics for the treatment of infections caused by such strains, due to their excellent pharmacokinetic properties and ability to penetrate various tissues [6,7]. However, the widespread use of these antibiotics has led to the development of MLS_B_-resistant staphylococci strains. Different molecular mechanisms have been observed that confer resistance to these antibiotics. Resistance, due to target site modification, is mainly mediated by genes of *erm* type (*ermA, ermB, ermC*), that are either constitutively (cMLS_B_ phenotype) or inducibly (iMLS_B_ phenotype) expressed. The *erm* genes code for the methylase enzyme, which methylates and alters the target site of MLS_B_ antibiotics, i.e., 23S ribosomal RNA. Resistance due to active efflux encoded by *msr* gene confer resistance to macrolides and streptogramin B (MS phenotype) but not to clindamycin [8,9]. The inducible clindamycin is responsible for therapeutic failure of the infections caused by staphylococci treated with clindamycin, because while performing routine antimicrobial susceptibility testing, such resistant isolates cannot be detected if erythromycin and clindamycin are not kept adjacent to each other [9,10]. The Clinical and Laboratory Standards Institute (CLSI) recommends simple, easy and reliable double disc diffusion (D test) for detection of inducible clindamycin resistant isolates [11].

This study was carried out to determine the prevalence of inducible clindamycin resistance and methicillin resistance, along with rate of biofilm production, among clinical staphylococcal isolates in Nepal.

## 2. Methods

### 2.1. Bacterial Isolates

A total of 375 non-repetitive clinical isolates of staphylococci were recovered from different specimens collected from patients admitted to B & B Hospital, Gwarko, Lalitpur, and KIST Medical College and Teaching Hospital, Imadole, Lalitpur, Nepal from August 2017 to September 2018. Patients of all age groups and genders who provided written informed consent for their voluntary participation were included in the study. Ethical approval to conduct the study was obtained from Nepal Health Research Council (NHRC) and the Institutional Review Committees (IRC) of the respective hospitals. Clinical samples such as urine, blood, wound swab/pus and different tips were included in this study. For the identification of the staphylococcal strain, the isolates were grown on nutrient agar, blood agar and mannitol salt agar, and the colony morphology were observed. The creamy to yellow colored, glistening, opaque colonies, with ß or weak zone of hemolysis on blood agar, were subjected to Gram staining. Those positive to Gram reaction with cocci shape, showing typical bunch arrangements, were subjected to a series of biochemical tests. The isolates that are positive to catalase, methyl red, voges proskauer, fermentative, urease, nitrate reduction, alkaline phosphatase, gelatin hydrolyzing, lactose, mannitol, maltose, mannose, sucrose and trehalose fermenting were identified as staphylococci. The slide and tube coagulase, DNase production was used for differentiating *S. aureus* and CNS [12].

### 2.2. Antibiotic Susceptibility Test

Antibiotic susceptibility test was performed by modified Kirby Bauer disc diffusion method on Mueller Hinton agar (HiMedia India Pvt, Ltd., Bengaluru, India), following CLSI guidelines, using *S. aureus* ATCC 25923 as the control strain. The used antibiotic discs (HiMedia India Pvt. Ltd., Bengaluru, India) were penicillin g (10 units), cefoxitin (30 µg), ciprofloxacin (5 µg), clindamycin (2 µg), chloramphenicol (30 µg), erythromycin (15 µg), gentamicin (10 µg), tetracycline (30 µg) and cotrimoxazole (25 µg).

Those isolates with cefoxitin zone size ≥22 mm were considered methicillin-susceptible isolates and those with ≤21 mm were considered methicillin-resistant isolates [11].

### 2.3. Detection of Inducible Clindamycin-Resistant Strains

**Figure 1:**
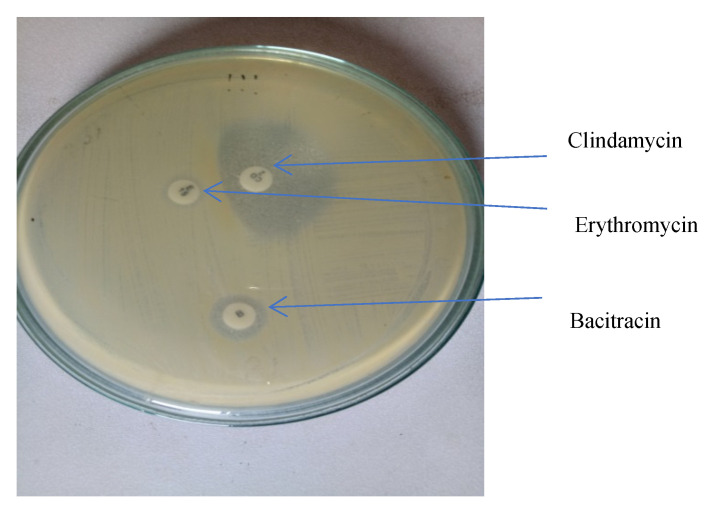
D zone test screening for inducible clindamycin-resistant staphylococci isolate no. 111.

Macrolide–lincosamide–streptogramin B (MLS_B_) resistance was detected by a double disc diffusion test, placing a 2 μg clindamycin disc 15 mm away from the edge of a 15 μg erythromycin disc on an MHA plate [11]. Following overnight incubation at 37 °C, 3 different phenotypes were appreciated and interpreted, as shown in Figure 1.

- Inducible MLS_B_ (iMLS_B_) phenotype—those isolates resistant to erythromycin but sensitive to clindamycin, showing a D-shaped zone of inhibition around clindamycin with flattening towards erythromycin disc.
- Constitutive MLS_B_ (cMLS_B_) phenotype—those isolates resistant to both erythromycin and clindamycin.
- MS phenotype—those isolates resistant to erythromycin and sensitive to clindamycin.

### 2.4. Detection of Biofilm Formation

The biofilm formation of the isolates was determined by tissue culture plate method [13]. Each isolate from fresh culture was inoculated in tryptone soy broth (TSB), supplemented with 1% glucose and incubated at 37 °C for 24 h. Using fresh medium, the cultures (adjusted to 0.5 McFarland standard) were diluted 1:100. Individual wells of sterile 96-well, flat-bottom polystyrene tissue culture plates (Tarsons Products Pvt. Ltd, Kolkata, India) were filled with 200 µL of the diluted cultures. *S. epidermidis* ATCC 35984 and sterile broth were used as positive and negative control, respectively. After incubation at 37 °C for 24 h, the contents of each well were removed by gentle tapping. The wells were washed with 0.2 mL of phosphate-buffered saline (pH 7.2) 4 times to remove free-floating planktonic bacteria. Biofilm, formed by bacteria, adherent to the wells were fixed by 2% sodium acetate and stained by crystal violet (1%). Excess stain was removed by washing with deionized water and plates were kept for drying. Optical density (OD) of stained adherent biofilm was obtained using a micro-ELISA autoreader at wavelength 570 nm. Biofilm production was categorized as negative/weak, moderate and strong depending on the OD values of adherent cells, as follows: an OD value < 0.120 was categorized as negative/weak, those with OD > 0.120 and <0.240 were regarded as moderate biofilm producers. An OD value > 0.240 was indicative of strong biofilm-producing bacterial strains [14,15].

### 2.5. DNA Extraction and Detection of ica Genes

The genomic DNA was extracted, as previously described [15], using a DNA extraction kit, following the manufacturer’s instructions (Thermo Fischer Scientific Inc., USA).

The sequences of *icaA* and *icaD* (accession number U43366) were taken from the GenBank sequence of the National Center for Biotechnology Information (NCBI) database. Primers specific for *icaA* and *icaD* were designed by the Primer3 program and were purchased from Solis Biodyne (TAG Copenhagen, Denmark). The primer used for the detection of *icaA* was forward 5′-TCTCTTGCAGGAGCAATCAA-3′ and reverse 5′-TCAGGCACTAACATCCAGCA-3′ primer, generating a product size of 188-bp. Similarly, for detection of *icaD*, 5′-ATGGTCAAGCCCAGACAGAG-3′, was used as a forward primer and 5′-CGTGTTTTCAACATTTAATGCAA-3′ was used as a reverse primer, with the product size of 198 bp.

Each PCR was performed in a final volume of 25 µL consisting of 12 μL of master mix, 1 μL of each *icaA* and *icaD* forward and reverse primers, respectively, 8 μL RNase free water and 3 μL of extracted DNA. DNA amplification was carried out with the following parameters: preheating at 95 °C for 5 min, 35 cycles of amplification with denaturation at 94 °C for 30 s, annealing at 55 °C for 30 s and extension at 72 °C for 30 s, followed by a final extension at 72 °C for 2 min. The PCR products were analyzed by electrophoresis on 2% agarose gel, stained with SYBR safe (Invitrogen Bioservices India Pvt. Ltd., Bengaluru, India) dye.

### 2.6. Statistical Analysis

The statistical analysis was performed using SPSS 17.0 (SPSS Inc., Chicago, IL, USA) software. Odds ratios (OR) and 95% confidence intervals (95% CI) were calculated and *p* ˂ 0.05 was considered statistically significant.

## 3. Results

A total of 375 staphylococci were isolated from different samples and processed, with 161 (42.9%) and 214 (57.1%) being identified as *S. aureus* and CNS, respectively (Figure S1). *S. aureus* were most frequently isolated from wound/pus (*w/p*) samples (135, 36%), followed by central venous catheter (CVC) tip sampled (9, 2.4%). Similarly, CNS were isolated more frequently from blood (82, 21.9%) and w/p (56, 14.9%).

### 3.1. Antibiotic Susceptibilities of Staphylococcal Isolates

The majority of isolates were susceptible to commonly used antibiotics as tetracycline (90.4%) and chloramphenicol (93.9%). *S. aureus* was found to be highly resistant towards penicillin (95%) followed by cefoxitin (81.4%) and erythromycin (78.9%). Similarly, CNS was more resistant towards penicillin (91.6%) and erythromycin (72.4%). Multidrug resistance (MDR) phenotype was observed in 148 (91.4%) *S. aureus* and 159 (74.6%) CNS (Table 1). Among 375 staphylococcal isolates, 131 (34.9%) and 143 (38.1%) isolates were identified as methicillin-resistant *S. aureus* (MRSA) and methicillin-resistant CNS (MRCNS) by cefoxitin disc diffusion assay, respectively (Table 1).

### 3.2. Inducible Clindamycin Resistance among Staphylococci

Among 161 *S. aureus* isolates, 127 (78.9%) were resistant to erythromycin and 145 (90.1%) were sensitive to clindamycin. Both erythromycin and clindamycin resistance were observed among 17 (10.6%) isolates indicating constitutive MLS_B_ phenotype. D test was found positive for 56 (34.8%) isolates indicating inducible MLS_B_ phenotype; whereas, 57 (35.4%) showed true sensitivity to clindamycin, as they were D test negative, having the MS phenotype. The susceptible phenotype, erythromycin sensitive and clindamycin sensitive (E-S, CD-S), was exhibited by 33 (19.3%) isolates (Table 2).

Similarly, among 214 CNS, cMLS_B,_ iMLS_B_ and MS phenotypes were observed in 60 (28.0%), 31 (14.5%) and 70 (32.7%) isolates, respectively (Table 2).

Higher percentages of iMLS_B_ were found among *S. aureus* than CNS; contrastingly, higher cMLS_B_ were observed among CNS than *S. aureus*, which were significantly different (Table 2).

Among 131 MRSA, the constitutive and inducible phenotype was detected in 15 (11.4%) and 42 (32.1%) isolates, respectively, while in 30 MSSA (methicillin-sensitive *Staphylococcus aureus*), the constitutive phenotype was found in 2 (6.7%) isolates and the inducible phenotype was found in 14 (46.7%) isolates, respectively. The constitutive phenotypes were 1.26 times greater among MRSA than MSSA (*p* = 0.052, OR 1.26, 95% CI 1.16–1.37) but inducible phenotypes were 3.18 times greater among MSSA than MRSA (*p* = 0.034, OR 3.18, 95% CI 1.04–9.68) (Table 3).

Constitutive and inducible MLS_B_ phenotypes were found in 51 (35.7%) and 25 (17.5%) isolates among methicillin-resistant CNS, whereas they were found in 9 (12.7%) and 6 (8.5%) in methicillin susceptible CNS isolates, respectively. The constitutive MLS_B_ phenotype was predominant over the inducible resistant among MRCNS as compared with MSCNS (methicillin-resistant, coagulase-negative staphylococci) (Table 3).

When statistically compared, the constitutive MLS_B_ phenotype was determined to be 4.11 times greater (*p* ≤ 0.001, OR 4.12, 95% CI 1.82–9.25), and the inducible resistance phenotype 1.97 times greater (*p* = 0.157, OR 1.97; 95% CI 0.76–5.10), than that in methicillin-susceptible CNS isolates (Table 3).

### 3.3. Detection of Biofilm Formation by Phenotypic and Genotypic Methods

In vitro biofilm production was examined by standard TCP method and found that, among 174 (46.4%) isolates, *S. aureus* (84, 22.4%) and CNS (90, 24.0%) isolates were positive for biofilm formation. There was no significant difference in in vitro biofilm production between methicillin-resistant and methicillin-susceptible strains (Table 4, Figure S2).

Since *icaA* and *icaD* genes are associated with biofilm formation, we sought to examine the presence of these genes in all our isolates. The amplification of both genes was observed in 86 (22.9%) isolates—45 (12%) *S. aureus* and 41 (10.9%) CNS isolates, respectively. The presence of *ica* gene is statistically significant among methicillin-resistant isolates (Table 4, Figure S3).

The biofilm formation was detected among 138 staphylococci isolates by TCP method even though they were devoid of *ica* genes. Similarly, 50 isolates possessed *ica* genes but were found to be non-producers of biofilm through the phenotypic method.

### 3.4. Inducible Resistant Phenotype among Biofilm Producing Isolates

Among 174 isolates that were found to be biofilm producers through the TCP method, 35 (20.1%) showed constitutive MLS_B_ phenotypes and 27 (15.5%) showed inducible MLS_B_ phenotypes. Those isolates which were not found to produce biofilm by the TCP method showed constitutive and inducible MLS_B_ phenotypes in 41 (20.4%) and 49 (24.4%) isolates, respectively (Table 5).

The *ica* genes were detected among 86 isolates. Among these isolates, 11 (12.8%) and 19 (22.1%) showed constitutive and inducible MLS_B_ phenotypes, respectively. However, 61 (21.1%) and 56 (19.4%) isolates showed constitutive and inducible MLS_B_ phenotypes that were negative for *ica* genes, respectively (Table 5).

There was no significant difference between inducible clindamycin resistance with biofilm formation, as detected by the TCP method and by the presence of *ica* genes (Table 5).

Interestingly, inducible MLS_B_ was detected 1.71 times more frequently in non-biofilm producers than in biofilm producers (*p* = 0.043, OR = 1.71, 95% CI = 1.01–2.88). Similarly, constitutive MLS_B_ was 1.6 times more frequently detected in *ica* gene-negative isolates than those possessing the *ica* gene (*p* = 0.210, OR = 1.66, 95% CI = 0.75–3.68).

## 4. Discussion

The presence of staphylococci, especially those strain which generate an extracellular slime and constitute a biofilm, make clinical treatment extremely difficult. Besides enabling bacterial colonization of host tissues, it also prevents clearance of the bacteria by antimicrobial agents and host immune responses, leading to morbidity and mortality owing to the metastatic spread of abscesses [14,16]. The multidrug resistance among such staphylococci is an increasing problem, leading to difficulties in the treatment of staphylococci infection [17].

The isolates were found to be 90% susceptible to commonly used antibiotics, such as tetracycline and chloramphenicol. Resistance rate to other tested antibiotics (except penicillin, ciprofloxacin, cefoxitin, erythromycin and gentamicin) fared below 30%, which is lower than the resistance reported by various authors from Nepal [18,19,20]. Such differences observed in resistance rates might be due to differences in study settings. However, the notoriety of staphylococci in developing resistance to therapeutic agents has been known since the advent of penicillin resistance, particularly in response to the selective antibiotic pressure [21]. A high proportion of isolates (349, 93.1%) were resistant to penicillin in this study. The result was in accordance with a study by Ansari et al. [19], who reported 94.7% resistant isolates. This was expected, as it has been recognized that only a small proportion of the staphylococci lineages do not produce beta-lactamases [19].

Today, the concern surrounding methicillin resistance has reached a pinnacle. It is noteworthy that methicillin-resistant strains can cause both community- and hospital-acquired infections. Resistance to methicillin in staphylococci is primarily mediated by the presence of penicillin-binding protein 2a, encoded by the *mecA* gene. Prior antibiotic use is the most common risk factor for colonization and infection with methicillin-resistant strains. In our study, 81.4% and 66.8% represented MRSA and MRCNS, respectively, which is higher than results reported by Ansari et al. [19] (43.1%). The incidence of MRSA was reported to be 20% in 2001, 15.4% in 2005, 26.14% in 2008, 39.6% in 2010 and 42.4% in 2013 in Nepal [20]. MRSA is a global phenomenon with a prevalence rate ranging from 2% in The Netherlands and Switzerland to 70% in Japan and Hong Kong [21]. Higher isolation rates reported in these studies can be attributed to several factors. These include indiscriminate use of antibiotics, lack of awareness and failure to observe simple yet effective infection-control precautions, such as strict patient isolation and frequent hand washing by health care personnel, population and in the area studied, etc. [20].

In this study, the rate of erythromycin resistance was higher than that reported by many authors [18,19,22,23] from Nepal, indicating wide prevalence of macrolide resistance among staphylococci and higher probability of such strains exhibiting inducible clindamycin resistance. Macrolide-resistant isolates of staphylococci may have constitutive or inducible resistance to clindamycin (due to methylation of the 23S rRNA encoded by the *erm* gene, also referred to as MLS_B_, or may be resistant only to macrolides (due to efflux mechanism encoded by the *msrA* gene).

We detected inducible clindamycin resistance in 87 (23.2%) of staphylococci isolates, which is slightly higher than results in previous studies, which reported resistance among 12.4%–22.4% of cases in Nepal [19,24,25]. In our study, the MS phenotype and constitutive MLS_B_ phenotype was higher among MRSA (41.2% and 11.4%) when compared with MSSA (10.0% and 6.7%). In contrast, previous studies [8] showed higher MLS_B_ phenotypes in MSSA as compared with the MRSA strains. The higher incidence of MLS_B_ in our study can be explained based on differences in various influencing factors, including the population studied, the geographical distribution, the health care facilities and the prevalence of MRSA and MSSA in the particular studied epidemiological area [8]. Among all MRCNS isolates, constitutive MLS_B_ were present among 51 (35.7%) isolates and inducible MLS_B_ were present among 25 (17.5%) isolates. Although a similar frequency of inducible MLS_B_ was demonstrated by Perez et al. [26], as high as 50% CNS were found to be positive in a study by Schreckenberger et al. [8]. These results indicate that constitutive and inducible MLS_B_ resistance varies depending on the hospital, the particular geographical area and the circulatory clones.

Clindamycin is frequently prescribed for the treatment of staphylococcal infections, including MRSA, and to those patients allergic to penicillin. However, there is risk of inducible resistance against clindamycin in patients, despite the in vitro susceptibility result. As the presence of an *erm* gene encoding for inducible resistance may result in treatment failure [10], it is important to perform its testing in routine laboratory analysis that would be beneficial for the detection of inducible clindamycin resistance among staphylococci strains for effective clinical prescription, hence, minimizing treatment failures.

Pathogenesis of staphylococci is attributed to a number of virulence factors, among which, biofilm formation is thought to be the most important one. Both phenotypic and genotypic methods were used to analyze the ability of biofilm production in all isolates. The TCP method is a convenient and quantitative technique that directly detects the polysaccharide production by measuring the adherent biofilm by spectrophotometer. Biofilm was detected in 84 (52.2%) *S. aureus*, with 70 (53.4%) MRSA. This result is consistent with the previous studies as presented by Mathur et al. [14], with 53.9%, and Knobloch et al. [27], with (64.7%). Among CNS, biofilm was detected in 90 (42.1%) isolates by TCP method with 58 (40.6%) isolates resistant to methicillin. A similar frequency of biofilm production in clinical CNS isolates was reported in previous studies: 43.9% was reported by Zhou et al. [28], 45% was reported by Cafiso et al. [29], 47.2% was reported by Prasad et al. [30] and 48.5% was reported by Aricola et al. [31]. However, the highest frequency (65.38%) of biofilm production was reported by Shrestha et al. [32] in a study conducted in a tertiary care hospital in eastern Nepal, which was similar to the study by Oliviera and Cunha [33], who reported that 75% of CNS are able to produce biofilm.

In the present study, concomitant presence of *icaA* and *icaD* genes was detected in 86 (22.9%) staphylococcal isolates, comprising 45 (28%) *S. aureus* and 41 (19.2%) CNS isolates. Previous studies have also demonstrated the presence of *ica* genes in clinical staphylococcal isolates. Los et al. [34] showed the prevalence of *ica* operon in 27.4% nasopharyngeal *S. epidermidis* isolates from hospitalized patients. Oliviera and Cunha [33] detected *ica* genes in 40% CNS isolated from clinical specimens and nares of healthy individuals. Likewise, Cafiso et al. [29] found that 35% of the isolates were positive for *icaA* and *icaD* genes; Nasr et al. [35] and de Silva et al. [36] showed that 32% and 40% staphylococcal isolates were positive for *ica* genes, respectively. We observed the presence of *ica* genes in *S. aureus*, accounting for 28% of the total staphylococcal isolates, which was slightly higher than in the CNS isolates. Biofilm formation has been observed in presence or absence of *ica* genes because there is evidence of PIA-independent biofilm formation.

In our study, higher rates of methicillin resistance were found among biofilm-producing strains in comparison with non-biofilm producing strains. These findings were in favor of the results reported by Ghasemian et al. [25]. Due to the protective nature of the biofilm, the bacteria growing in it are intrinsically resistant to many antibiotics. Furthermore, biofilm formation gives a platform for horizontal gene transfer among bacteria, causing the spread of drug resistance markers as MDR genes and other virulence genes, which limits therapeutic options.

A limited study has been carried out regarding correlation of biofilm formation with inducible clindamycin resistance in staphylococci. There was no significant difference found between inducible clindamycin resistance phenotypes and biofilm formation. More studies are required to assess the relationship, as biofilm development is a very complicated process that involves numerous factors. Similarly, to understand the molecular mechanism, the detection and analysis of several genes, such as *erm* and *msr*, are essential.

## 5. Conclusions

Staphylococci are emerging as a major health problem due to multidrug resistance and biofilm formation. Clindamycin resistance in the form of iMLS_B_ and cMLS_B_ limits the therapeutic options for methicillin-resistant staphylococci. The present study revealed that MRSA strains, isolated from clinical samples, produced biofilm and possessed *icaA* and *icaD* genes. A significant correlation was not found between biofilm formation and inducible clindamycin resistant phenotypes among the studied staphylococci isolates. Further investigation of resistant genes is necessary for better understanding of this relationship.

## Figures and Tables

**Table 1:** Antimicrobial susceptibility profiles of staphylococci.

Antimicrobial Agent	Antimicrobial Class	*S. aureus* (*n* = 161)		CNS (*n* = 214)		Total (*n* = 375)	
		Sensitive *n* (%)	Resistant *n* (%)	Sensitive *n* (%)	Resistant *n* (%)	Sensitive *n* (%)	Resistant *n* (%)
Penicillin	β lactams	8 (5%)	153 (95%)	18 (8.4%)	196 (91.6%)	26 (6.9%)	349 (93.1%)
Ciprofloxacin	Fluroquinolone	42 (26.1%)	119 (73.9%)	138 (64.5%)	76 (35.5%)	180 (48%)	195 (52%)
Tetracycline	Tetracycline	152 (94.4%)	9 (5.6%)	187 (87.4%)	27 (12.6%)	339 (90.4%)	36 (9.6%)
Clindamycin	Lincosamide	145 (90.1%)	16 (9.9%)	151 (70.6%)	63 (29.4%)	296 (78.9%)	79 (21.1%)
Chloramphenicol	Phenicols	157 (97.5%)	4 (2.5%)	195 (91.1%)	19 (8.9%)	352 (93.9%)	23 (6.1%)
Cefoxitin	β lactams	30 (18.6%)	131 (81.4%)	71 (33.2%)	143 (66.8%)	101 (26.9%)	274 (73.1%)
Erythromycin	Macrolides	34 (21.1%)	127 (78.9%)	59 (27.6%)	155 (72.4%)	93 (24.8%)	282 (75.2%)
Cotrimoxazole	Folic acid synthesis inhibitors	71 (44.1%)	90 (55.9%)	134 (62.6%)	80 (37.4%)	205 (54.7%)	170 (45.3%)
Gentamicin	Aminoglycosides	94 (58.4%)	67 (41.6%)	169 (79%)	45 (21%)	263 (70.1%)	112 (9.9%)

**Table 2:** Inducible clindamycin-resistant phenotypes in *S. aureus* and CNS.

Phenotypes	*S. aureus* (*n*, %)	CNS (*n*, %)	Total (*n*, %)	*p* Value	OR	95% CI
E-S, CD-S (susceptible)	31 (19.3%)	53 (24.8%)	84 (22.4%)	0.303	0.77	0.47–1.26
E-R, CD-R (cMLS_B_)	17 (10.6%)	60 (28.0%)	77 (20.5%)	≤0.001	0.18	0.08–0.41
E-R, CD-S, D^+^ (iMLS_B_)	56 (34.8%)	31 (14.5%)	87 (23.2%)	0.046	0.54	0.29–0.99
E-R, CD-S, D^−^ (MS phenotype)	57 (35.4%)	70 (32.7%)	127 (33.9%)	≤0.001	2.70	1.60–4.55
Total	161 (100%)	214 (100%)	375 (100%)			

E—erythromycin; CD—clindamycin; S—sensitive; R—resistant; D^−^—D test negative; D^+^—D test positive; cMLS_B_—constitutive MLS_B_; iMLS_B_—inducible MLS_B_; MLS_B_—macrolide–lincosamide–streptogramin B; MS—macrolide–streptogramin B; OR—odds ratio; CI—confidence interval.

**Table 3:** Prevalence of inducible clindamycin resistance phenotype among methicillin-resistant staphylococci.

Phenotype	MRSA *n* (%)	MSSA *n* (%)	*p* Value	OR	95% CI	MRCNS *n* (%)	MSCNS *n* (%)	*p* Value	OR	95% CI	Total *n* (%)
E-S, CD-S (susceptible)	20 (15.3%)	11 (36.7%)	0.001	0.24	0.09–0.56	18 (12.6%)	35 (49.3%)	≤0.001	0.15	0.08–0.29	84 (22.4%)
E-R, CD-R (cMLS_B_)	15 (11.4%)	2 (6.7%)	0.052	1.26	1.16–1.37	51 (35.7%)	9 (12.7%)	≤0.001	4.11	1.82–9.25	77 (20.5%)
E-R, CD-S, D^+^ (iMLS_B_)	42 (32.1%)	14 (46.7%)	0.034	3.18	1.04–9.68	25 (17.5%)	6 (8.5%)	0.157	1.97	0.76–5.10	87 (23.2%)
E-R, CD-S, D^−^ (MS phenotype)	54 (41.2%)	3 (10.0%)	0.714	0.86	0.39–1.92	49 (34.3%)	21 (29.6%)	0.318	1.37	0.74–2.55	127 (33.9%)
Total	131 (34.9%)	30 (8.0%)				143 (38.1%)	71 (18.9%)				375

**Table 4:** Methicillin resistance and biofilm formation of staphylococcal isolates.

Method	Biofilm Formation	MRSA (131)	MSSA (30)	*p* Value	MRCNS (143)	MSCNS (71)	*p* Value	Total (375)
TCP	Positive	70 (53.4%)	14 (46.7%)	0.681	58 (40.6%)	32 (45.1%)	0.412	174 (46.4%)
	Negative	61 (46.6%)	16 (53.3%)		85 (59.4%)	39 (54.9%)		201 (53.6%)
*ica* genes	Positive	29 (22.1%)	16 (53.3%)	0.001	28 (19.6%)	13 (18.3%)	0.824	86 (22.9%)
	Negative	102 (77.9%)	14 (46.7%)		45 (31.5%)	58 (81.7%)		289 (7.1%)

**Table 5:** Inducible clindamycin resistance and biofilm formation.

Phenotypes	Biofilm Detection									
	TCP					*Ica*				
	Positive	Negative	*p* Value	OR	95% CI	Positive	Negative	*p* Value	OR	95% CI
E-S, CD-S (susceptible)	43 (24.7%)	44 (21.9%)	0.446	0.83	0.51–1.34	31 (36.0%)	55 (19.0%)	0.001	0.42	0.25–0.71
E-R, CD-R (cMLS_B_)	35 (20.1%)	41 (20.4%)	0.503	1.23	0.67–2.24	11 (12.8%)	61 (21.1%)	0.210	1.66	0.75–3.68
E-R, CD-S, D^+^ (iMLS_B_)	27 (15.5%)	49 (24.4%)	0.043	1.71	1.01–2.88	19 (22.1%)	56 (19.4%)	0.580	0.85	0.47–1.52
E-R, CD-S, D^−^ (MS phenotype)	69 (39.7%)	67 (33.3%)	0.66	1.14	0.64–2.03	25 (29.1%)	117 (40.5%)	0.575	1.22	0.60–2.49
Total	174 (46.4%)	201 (53.6%)				86 (22.9%)	289 (7.1%)			

## Data Availability

All the data pertaining to this study are within the manuscript.

## Supplementary Materials

The following are available online at https://www.mdpi.com/article/10.3390/idr13040095/s1, Figure S1 (a). Growth of Coagulase negative Staphylococci (CNS) and *Staphylococcus aureus* in Mannitol salt agar media; (b). Growth of Staphylococci isolates in Blood agar media; Figure S2. Detection of biofilm production by Tissue Culture Plate Method; Figure S3. *icaA* and *icaD* gene amplified product after PCR in agarose gel electrophoresis.
