# Supplementary material for: Inducible Clindamycin Resistance and Biofilm Production among Staphylococci Isolated from Tertiary Care Hospitals in Nepal
Source: Infect Dis Rep. 2021 Dec 7;13(4):1043–52. doi: 10.3390/idr13040095 (PMC8702181; doi:10.3390/idr13040095)
Supplement: Supplementary file 1 [file idr-13-00095-s001.zip › idr-1451783-supplementary.pdf]

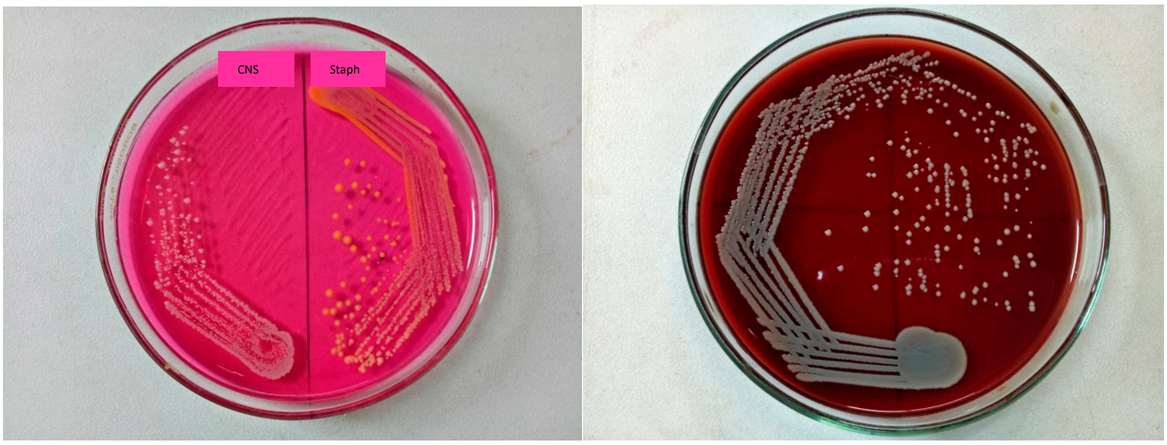


(a) (b)

Figure S1(a). Growth of Coagulase negative Staphylococci (CNS) and *Staphylococcus aureus* in Mannitol salt agar media; (b). Growth of Staphylococci isolates in Blood agar media

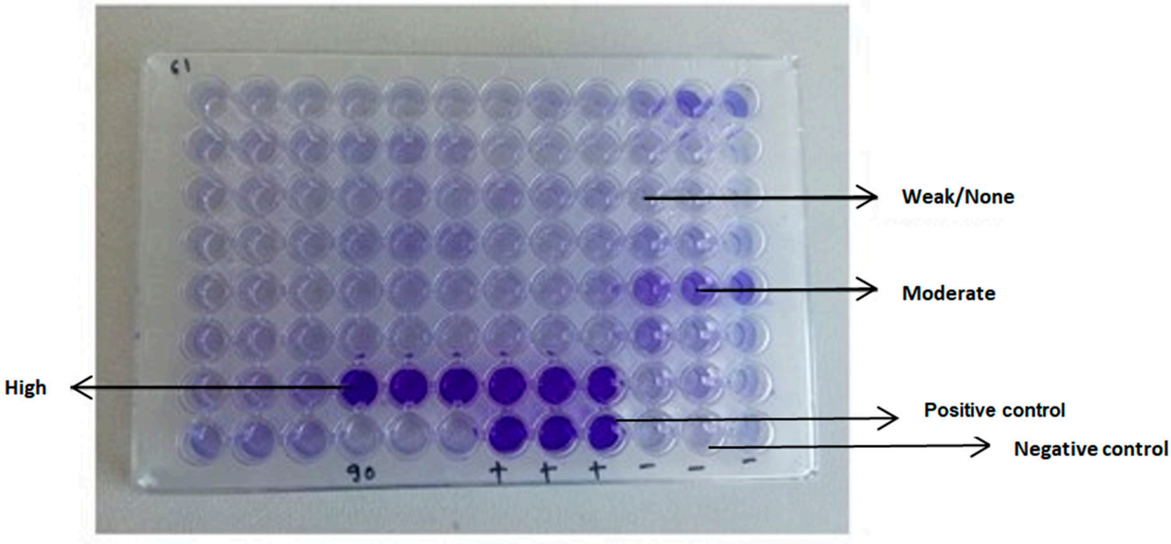


Figure S2. Detection of biofilm production by Tissue Culture Plate Method

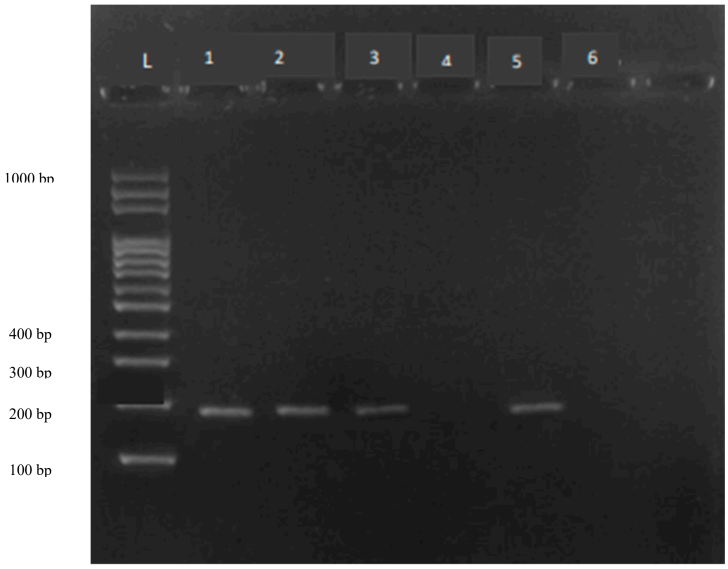


Figure S3 *icaA* and *icaD* gene amplified product after PCR in agarose gel electrophoresisL: 1 Kb DNA Ladder; Lane 1: positive control for *icaA*; Lane 2:positive control for *icaD*; Lane 3: sample positive for *icaA*; Lane 4: Blank; Lane 5: sample positive for *icaD*; Lane 6: sample negative for both genes
